# Gametogenesis in the Pacific Oyster *Crassostrea gigas*: A Microarrays-Based Analysis Identifies Sex and Stage Specific Genes

**DOI:** 10.1371/journal.pone.0036353

**Published:** 2012-05-09

**Authors:** Nolwenn M. Dheilly, Christophe Lelong, Arnaud Huvet, Kristell Kellner, Marie-Pierre Dubos, Guillaume Riviere, Pierre Boudry, Pascal Favrel

**Affiliations:** 1 Université de Caen Basse-Normandie, “Biologie des Organismes Marins et des Ecosystèmes Associés ”(BioMEA), IBFA, SFR ICORE, Caen, France; 2 CNRS INEE, BioMEA, Caen, France; 3 Ifremer, Laboratoire des Sciences de l’Environnement Marin, Centre de Bretagne, Plouzané, France; University of Maryland School of Medicine, United States of America

## Abstract

**Background:**

The Pacific oyster *Crassostrea gigas* (Mollusca, Lophotrochozoa) is an alternative and irregular protandrous hermaphrodite: most individuals mature first as males and then change sex several times. Little is known about genetic and phenotypic basis of sex differentiation in oysters, and little more about the molecular pathways regulating reproduction. We have recently developed and validated a microarray containing 31,918 oligomers (Dheilly et al., 2011) representing the oyster transcriptome. The application of this microarray to the study of mollusk gametogenesis should provide a better understanding of the key factors involved in sex differentiation and the regulation of oyster reproduction.

**Methodology/Principal Findings:**

Gene expression was studied in gonads of oysters cultured over a yearly reproductive cycle. Principal component analysis and hierarchical clustering showed a significant divergence in gene expression patterns of males and females coinciding with the start of gonial mitosis. ANOVA analysis of the data revealed 2,482 genes differentially expressed during the course of males and/or females gametogenesis. The expression of 434 genes could be localized in either germ cells or somatic cells of the gonad by comparing the transcriptome of female gonads to the transcriptome of stripped oocytes and somatic tissues. Analysis of the annotated genes revealed conserved molecular mechanisms between mollusks and mammals: genes involved in chromatin condensation, DNA replication and repair, mitosis and meiosis regulation, transcription, translation and apoptosis were expressed in both male and female gonads. Most interestingly, early expressed male-specific genes included *bindin* and a *dpy-30* homolog and female-specific genes included *foxL2*, *nanos homolog 3*, a *pancreatic lipase related protein*, *cd63* and *vitellogenin.* Further functional analyses are now required in order to investigate their role in sex differentiation in oysters.

**Conclusions/Significance:**

This study allowed us to identify potential markers of early sex differentiation in the oyster *C. gigas*, an alternative hermaphrodite mollusk. We also provided new highly valuable information on genes specifically expressed by mature spermatozoids and mature oocytes.

## Introduction

*C. gigas* which belongs to the Lophotrochozoa, the largely understudied third clade of bilaterian animals, represents a non-model species for which the genomic resource available turns out to be very substantial [1], [2]. This offers a unique opportunity to investigate structure/function shifts during evolution and, by comparison with data from the two other bilaterian clades, to help define the basic assortment of genes required to manage reproduction.

From a more applied point of view, understanding the mechanisms regulating the seasonal reproductive cycle of *C. gigas* is of economical relevance. Indeed, the Pacific oyster is one of the most important species in aquaculture worldwide. Hatchery production of oyster seed is expanding significantly, as an alternative to traditional aquaculture methods relying on natural seed collection, allowing the development of selective breeding programs [3]. Another key interest for aquaculture is the production of triploid oysters that show faster growth and higher market value due to their reduced gonad development [4], [5]. Thus, aquacultural production may benefit from a better knowledge of the genes involved in reproduction [6], [7].

Like many marine invertebrates, Pacific oysters have a very high fecundity, characterizing the “r”-selected strategy. Evolution has shaped the physiology of these species to optimize their fitness by increasing their allocation to reproduction. As a result, gametogenesis has a major impact on several physiological functions. This impact can be revealed by studying genetic and phenotypic tradeoffs between fitness-related traits [8]. In oysters, gametogenesis is a period of negative energy balance due to the high production of gametes [9], [10]. This critical period has been shown to be detrimental for defense mechanisms [11], [12]. More specifically, the end of the maturation period appears to be correlated with summer mortality [13] one of the current major concern of oyster aquaculture in the world. Interestingly, lines selected for susceptibility (S) or resistance (R) to summer mortality, a highly heritable trait in *Crassostrea gigas* [14], [15], [16], [17] show different investments in reproduction. R families display lower reproductive effort than S families thus suggesting that R lines survive summer mortality because they are less reproductively active than S lines [15], [18].

*C. gigas* is an irregular successive hermaphrodite, generally protandrous in the first year Most individuals first develop as males [19] and then can change sex from one reproductive season to the other, resulting into labile population sex ratios. Synchronous hermaphrodites can also seldom be observed [20]. Its gonad is a mixed tissue including storage tissue, smooth muscle fibers and circulating hemocytes surrounding the digestive system. The primary gonad of *C. gigas* contains germ cells that derive from germinal stem cells produced by early differentiation of primordial germ cells during embryogenesis [21]. During the initial stage of gametogenesis (Stage 0), small clusters of self-renewing stem cells appeared scattered in conjunctive tissue [21], [22]. At this stage, the sex of an individual cannot be determined, even by histological observations. During stage 1, germ cells divide by mitosis and differentiate to produce a large number of gonia (gonial proliferation) [22], [23], [24], [25], [26], [27]. From this stage, the sex of individuals can be determined using histological methods. The mitotic activity of the cells induces the expansion of tubules that invaginate the storage tissue surrounding the digestive system of oysters. Gonads classified in stage 2 have maturating germ cells in developing gonadic tubules that grow and ramify at the expense of the storage tissue [25], [28], [29]. In stage 3, gonads are fully mature and completely filled due to the confluence of gonadic tubules [23], [24], [25].

As a result, the gonad in *C. gigas* is a diffuse and non permanent tissue composed of somatic cells and germ cells that surrounds the digestive gland. Spawning commonly occurs during spring or summer under temperate climate as reproduction is mainly induced by temperature and food availability [24]. The current understanding of the signaling pathways implicated in gonad differentiation and development in oysters is limited to a few genes (for example *Cg-foxL2*, *PY-PLRP*, *Cg-DMl*, *bindin*, *vasa*) [21], [30], [31], [32], [33], [34], [35]. The understanding of sex differentiation, the regulation of gonad development *versus* storage tissue, and spawning is very limited despite being a critical issue in both fundamental and applied contexts.

Therefore, this study was designed to provide a better understanding of the molecular mechanisms underlying the course of a reproductive cycle of male and female oysters by describing their gonad transcriptome, and to establish lists of genes of interest specific to each reproductive stage and sex. We employed a custom oligonucleotide microarray containing 31,918 ESTs described and validated in Dheilly et al. [2]. Our study identifies novel sex specific molecular markers and genes differentially expressed over the different stages of the gametogenesis cycle of males and females.

## Results

In order to provide a global view of the transcriptional changes that occur during male and female gonad development from the undifferentiated gonad stage (stage 0) to the fully mature gonad (stage 3), the four successive developmental stages commonly distinguished (see Material and Methods) were studied. Before microarray analysis, sex and gametogenetic stages were determined histologically [24], [25]. The transcriptome of 32 individual gonads representing these 4 gametogenetic stages and the 2 sexes was characterized. In addition, transcriptome of oocytes collected by stripping gonads of 7 females were compared to the transcriptome of full stage 3 female gonads to allow the localization of gene specific expression in germ cells or somatic cells. Finally, the transcriptomes of 6 pools of stage 3 females from different geographical locations were also described for biological validation of gene expression features. A total of 45 separate arrays were used and the complete dataset was made available through NCBI via the Gene Expression Omnibus (GEO) data repository (GSE 27955; http://www.ncbi.nlm.nih.gov/geo/query/acc.cgi?acc=GSE27955).

### Prevalent Gene Expression Patterns

**Figure 1:**
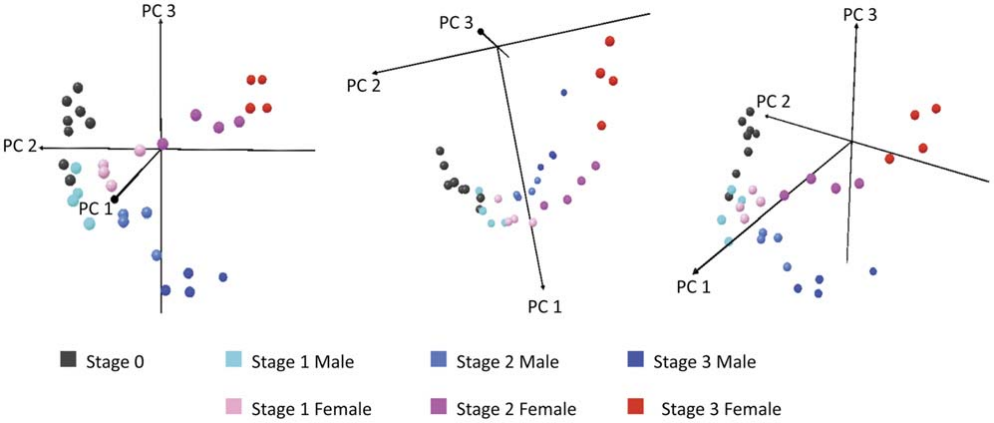
Principal component analysis. 3D Score plot using the first 3PCs identified by principal components analysis of all 31,918 transcripts in the 32 individual oyster gonads sampled from Site 1.

Principal Component Analysis (PCA) was applied to all 31,918 transcripts of the 32 oyster individuals from Site 1 (Locmariaquer, Brittany, France) to assess internal consistency of the whole transcriptional dataset and verify that the main variation in gene expression corresponds specifically to gonad developmental stages and sexes. The first three Principal components (PCs) explained 93.1% of the total variance. The 3D score plot obtained using the three first PCs is shown in Figure 1. In this plot, similar transcriptional profiles cluster together, whereas significantly different samples appear more distant from each other. We observed a clear clustering of the different gametogenetic stages determined by histological methods. Interestingly, gonad developmental stages were organized along PC2 with decreasing component loadings from stage 0 to stage 3. PC3 discriminated male and female individuals with low component loadings for males and high component loadings for females.

The organization of reproductive stages from the two sexes along a single PC (PC2) suggests that male and female gonads share similar expression profiles through their development. However, a significant divergence in expression patterns between male and female gonads was observed from stage 1 and throughout gametogenesis (PC3). At stage 3, males and females predictably possess the most distinctive expression profiles. As a result, gene expression patterns should allow determining with certitude the sex of an individual from stage 1 without histological information.

### Identification of Sex-specific Expressed Genes

**Figure 2:**
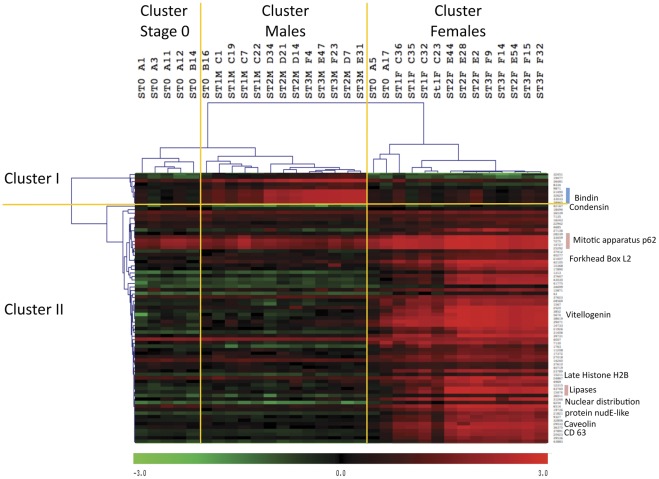
Heat map of sex specific genes. Hierarchical clustering obtained using Pearson’s correlation on the 77 genes differentially expressed in males and females (T test, p<0.01, adjusted Bonferroni’s correction, rows) and on all individual gonad samples (columns). Three sample branches are observed, mainly clustering stage 0 individuals, males, or females. Two gene branches are observed, clustering genes more expressed in males (cluster 1∶9 genes) or in females (cluster 2∶68 genes). Color represents the transformed normalized Cy3 log value obtained for each sample. The variations in transcript abundance are depicted with a color scale, in which shades of red represent higher gene expression and shades of green represent lower gene expression. St3: stage 3; St2: stage 2; St1: stage 1; St0: stage 0.

In order to identify sex-specific gene expression features, we realized student’s T tests (p<0.01 with adjusted Bonferroni’s correction) on all male *versus* all female gonads, regardless of their reproductive stages. This analysis identified 77 genes differentially expressed between the two sexes (file S1). Hierarchical clustering using Pearson’s correlation on individual gonads identified two clusters of coordinately regulated genes (Figure 2). Cluster I grouped 9 genes significantly over expressed in males compared to females, whereas cluster II grouped the remaining 68 genes significantly more expressed in females. Among these 9 genes significantly more expressed in males, 5 showed highly similar expression patterns [Genbank CF369228, EF219426, EF219427, EF219428, EF219429]. They correspond to *bindin* precursors 1, 2, 3, 4 and 5 repeat variants. Among the 68 genes significantly more expressed in females, we noticed the presence of the genes coding for Forkhead box protein L2 [Genbank AM860211], Vitellogenin [Genbank AB084783], Lipoprotein lipase [Genbank FP000833], Pancreatic lipase-related proteins [Genbank AM862314, AM857075], Mitotic apparatus protein p62 variants [Genbank CU987956, FP089842, AM237660], Tetraspanin (CD63) [Genbank EW779447], and Condensin 2 [Genbank CU993108].

Hierarchical clustering of individual gonad data identified three main clusters: Stage 0, Male, and Female. Stage 0 individuals appeared closer to males than to females, which fits with the much lower number of male-specific genes than female-specific genes. There were 3 exceptions to the clustering of stage 0 individuals: Individual B16 clustered on the same branch as males and individuals A5 and A17 clustered together with female gonads. Although the sex of these oysters could not be determined histologically, we suggest that these individuals had already initiated sex differentiation and could therefore be classified according to their gene expression pattern. Indeed, individual B16 highly expresses *bindin* and individuals A5 and A17 express the female-specific genes *foxL2*, *pancreatic lipase related protein*, *cd63*, and other non-annotated genes identified as female-specific in this study.

### Identification of Genes Whose Expression Changed During Gonad Development

**Figure 3:**
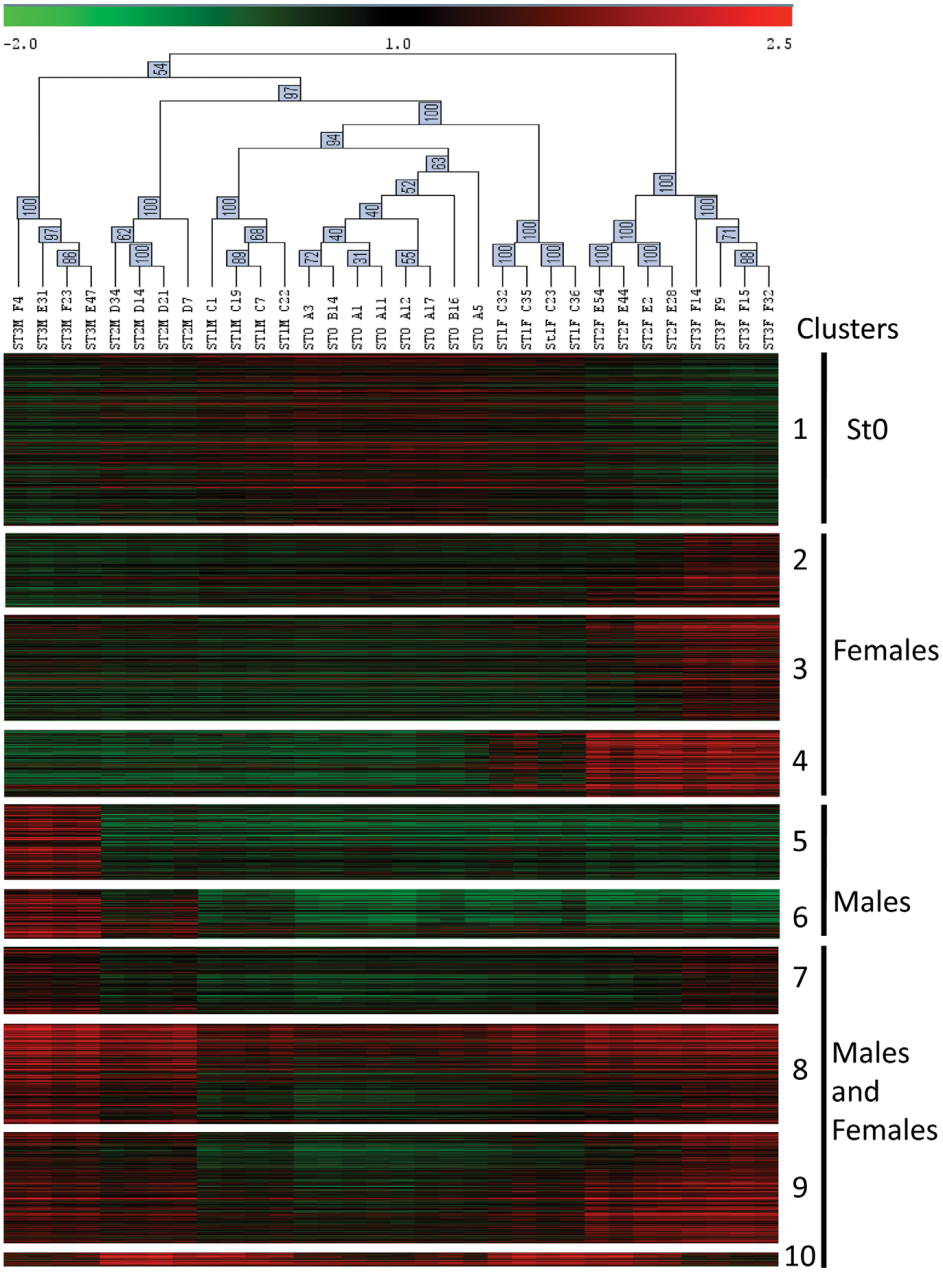
Heat map of clusters of genes differentially expressed during gametogenesis. Hierarchical clustering obtained using Pearson’s correlation on the 32 unique transcriptomic profiles of gonads (columns). Clusters of the 2,482 differentially expressed genes (rows) have been obtained with k means clustering using Pearson’s correlation. Individual gonad samples from the same developmental stage and sex grouped together. St3: stage 3; St2: stage 2; St1: stage 1; St0: stage 0. Genes showing similar expression profiles clustered together. Four main groups are identified: stage 0: genes more expressed at early gonadogenesis stages (stage 0 and stage 1); Males and females: genes that vary in expression over the course of both male and female gametogenesis; Male: genes that increase in expression over the course of spermatogenesis; Female: genes that increase in expression over the course of oogenesis. Color represents the transformed normalized Cy3 log value obtained for each sample. The variations in transcript abundance are depicted with a color scale, in which shades of red represent higher gene expression and shades of green represent lower gene expression.

One-way ANOVA (p<0.01, adjusted Bonferroni’s correction) identified 2,482 differentially expressed genes between gametogenetic stages (file S2). These genes exhibited a significant change in expression over the reproductive stages of male and/or female gonads. Hierarchical clustering using Pearson’s correlation grouped the 32 individual gonad samples according to their sex and their gonad developmental stage (Figure 3). K means clustering of genes using Pearson’s correlation produced 10 distinct clusters of genes with similar expression patterns (Figure 3). The lists of genes grouped within each cluster are provided in file S2. Biological interpretation of the data led us to group these clusters into 4 major gene expression profiles: (I) genes more expressed in gonads in early gonad developmental stages (stage 0 and stage 1; cluster 1), (II) genes with increasing expression over the course of spermatogenesis (clusters 5 and 6), genes with a significant shift in the expression pattern from stage 2 to stage 3, probably reflecting the maturation of spermatozoids, (III) genes with increasing expression over the course of oogenesis (clusters 2, 3 and 4) and (IV) genes with varying expression level over the course of gonadogenesis both in males and females (clusters 7, 8, 9 and 10).

Twice more genes were found differentially expressed over the course of oogenesis than during spermatogenesis. Among the 731 genes with increasing expression over the course of oogenesis, 197 were found expressed early in stage 1 (cluster 4), 312 appear in stage 2 (cluster 3) and 222 are expressed in mature stage 3 gonads only (cluster 2). Genes expressed from stage 1 included the previously identified female specific genes *vitellogenin*, *cd63*, *mitotic apparatus p62*, *forkhead box L2* and *caveolin*. Additional genes potentially involved in female germ cell differentiation were identified in this cluster including *nanos homolog 3* [Genbank CU988662 and CU987956] and *lsm14 homolog* [Genbank AM855747]. Genes illustrating oogenesis were found in the two other female specific clusters such as genes coding for the Protein strawberry notch homolog 1 [Genbank AM858733], Nucleosporin 53 [Genbank AM861011], Cep57 [Genbank FP002000], Storkhead box protein 1 [Genbank AM864470] and a membrane progestin receptor [Genbank AM861178].

Among the 372 genes with increasing expression along the process of spermatogenesis, 146 showed an increase in expression from stage 1 to stage 3 (cluster 6) and 226 were specific to mature spermatozoids found in stage 3 male gonads (cluster 5). Expressed early during spermatogenesis, we identified a gene potentially involved in sex differentiation, *dpy-30* [Genbank CU998852] and genes involved in meiosis such as *histone h4*, *meiotic recombination protein dmc1/lim15 homolog*, *synaptonemal complex protein 1*, and *sister chromatid cohesion protein pds5 homolog* [Genbank DW713865, EX956377, AM961984, CU988915]. In cluster 5, we identified numerous sperm specific genes such as *sperm associated antigens 6* and *8*, *motile sperm domain containing protein 2*, *sperm surface protein Sp17*, *sperm flagellar proteins 1* and *2* [Genbank AM860311, CU684841, CU990254, AM859254, AM858256, CU994483, AM864034, AM867566, AM857403].

Four clusters grouped genes involved in both male and female gametogenesis. Among them, cluster 10 was particularly peculiar as it groups 41 genes that increased in expression from stage 0 to stage 1 and 2 and decreased in stage 3. Cluster 10 grouped genes coding for proteins necessary for the organization of microtubule cytoskeleton during growth such as Filamins A and C [Genbank CU999039, FP000131, FP002575, CU685900, FP003903, FP004316], Talin [Genbank CU999937], Futsch [Genbank AM856252], or involved in muscle growth such as Kyphoscoliosis peptidase [Genbank CU991317]. Clusters 8 and 9 grouped the 868 genes that displayed an increase in expression during the time course of gametogenesis in both male and female oysters. The great majority were found expressed by germ cells rather than somatic cells in stage 3 females. They are associated with functions that are consistent with events known to occur at different stages of gametogenesis such as the regulation of chromatin condensation (*condensin-2, histone h3-like centromereic protein, histone h2a.v*), DNA replication control (*dna replication licensing factor, dna replication complex gins, replication factor c*), DNA repair (*dna mismatch repair protein msh6, dna ligase 1, bloom syndrome protein homolog, dna topoisomerase 2, dna double stranded break repair rad50 atpase, fanconi anemia associated protein*), mitosis and meiosis regulation (*centromere proteins, claspin, nude, spindle and kinetochore associated protein 1, cyclin dependent kinase and cyclin dependent kinase inhibitor, kinesin related motor protein, carboxy terminal kinesin, chromosome associated kinesin, targeting protein for xklp2, stathmin, nucleoporins, anillin, rhi GTPase activating protein, actin depolymerizing factor, protein regulator of cytokinesis*), regulation of transcription (*mortality factor 4-like protein 1, histone acetyltransferase myst1, kinetochore proteins*), translation (*nuclear factor of kappa light polypeptide, enhancer in b cell inhibitor, eukaryotic translation initiation factor, zinc finger protein 141, exoribonuclease, mediator of rna polymerase II transcription subunit, dna directed rna polymerase II subunit, poly adp-ribose polymerase 1*), and regulation of apoptosis (*ubiquitin conjugating enzyme e2, ubiquitin carboxyl-terminal hydrolase, leucine rich repeat and death domain containing protein, protein tumorous imaginal discs*). These are known to be involved in male and female germ cells development in various organisms from yeast to mammals [36]. All these genes constitute a list of candidate genes to further explore specific pathways implicated in reproduction of marine bivalves.

Genes grouped in cluster 7 were highly expressed in stage 3 males and females only. This cluster included the *polycomb protein suz12* [Genbank AM863465], *DNA (cytosine-5) methyltransferase* [Genbank CU999215, CU994437], *histone H4 transcription factor* [Genbank AM863339], and *histone acetyltransferase* [Genbank AM856009] potentially playing a role in epigenetic mechanisms. In addition, genes known to be involved in mitosis and meiosis regulation and cell cycle arrest were highly expressed in stage 3 individuals.

Five hundred and eleven genes decreased in expression along the gametogenetic cycle (Cluster 1). Numerous genes were previously identified as tissue-enriched in either the digestive gland, the mantle tissue, the visceral ganglion, hemocytes or the adductor muscle by Dheilly et al. [2]. The method we employed did not exclude the possibility that genes that appear more expressed in early gonad developmental stages than in maturing gonads could be artifacts due to a dilution effect of genes expressed in somatic tissues, such as muscular fibers surrounding the gonadal tubules, when germ cells accumulate within the gonad area. In order to identify genes specifically expressed during early gametogenetic stages, we searched for genes significantly more expressed in immature gonads than in somatic tissues and mature gonads. Thus we compared expression data in stage 0 oyster gonads with expression data from somatic tissues previously described in Dheilly et al. [2] in order to differentiate germline specific genes and genes somatically expressed in our gonad samples. Numerous genes characterizing muscle fibers were highly expressed in mantle and the adductor muscle including *calponin* [Genbank AM854381, CX739578], *myosin* [Genbank CU686207, CU683452, ES789928], *titin* [Genbank AM860424, CU685775, CU683161, CU684224, FP000452, FP002471], *tropomyosin* [Genbank CU686158], *unc-89* [AM858770] and *calmodulin* [AM854780]. Immune response genes were found expressed in oyster gonads and mantle only, including genes coding for the Complement C3, a PZP-like alpha-2-macroglobulin domain containing protein [Genbank CU990177], Small proline rich protein 3 [Genbank AM869022, AM860950, AM865427], Chitotriosidase [Genbank AJ971239], Superoxide dismutase [Genbank CX069299] and CD109 [Genbank CU988853]. Other genes were found highly expressed in either hemocytes, the visceral ganglia or in all somatic tissues (file S2). These results confirmed that genes in cluster 1 are mostly somatically expressed in mantle-gonad tissue.

In contrast, few genes whose expression increased over the course of gametogenesis were also highly expressed in key somatic tissues. Among the genes that increased in expression in both males and females, we observed a high expression of two genes coding for regulatory proteins of microtubule dynamics, *stathmin* and *stathmin-2* [Genbank AM866816 and AM853986] that were also highly expressed in the visceral ganglia. Among the genes that increased in expression during female gametogenesis, a unique gene, the *GTP binding protein RAD* [Genbank AM857574], was more expressed in muscle than in female gonad. However, among the genes only highly expressed in male gonads, as many as 151 genes expressed in stage 3 male gonads were also highly expressed in labial palps and gills. Further investigation of these predicted gene functions revealed that they may be involved in flagella and cilia structure, locomotion and control, such as genes coding for Kinesin-like protein [Genbank CI997201], Sperm surface protein Sp17 [Genbank AM864034], Sperm associated antigen 8 [Genbank AM859254], Sperm flagellar protein 2 [Genbank AM857403] and numerous Coiled coil domain containing proteins [Genbank CU685202, FD483996, AM856906, AM857430]. Genes highly expressed in stage 3 male gonads, labial palps and gills are listed in file S2.

### Identification of Oocyte-specific Genes *versus* Somatic Cells

**Figure 4:**
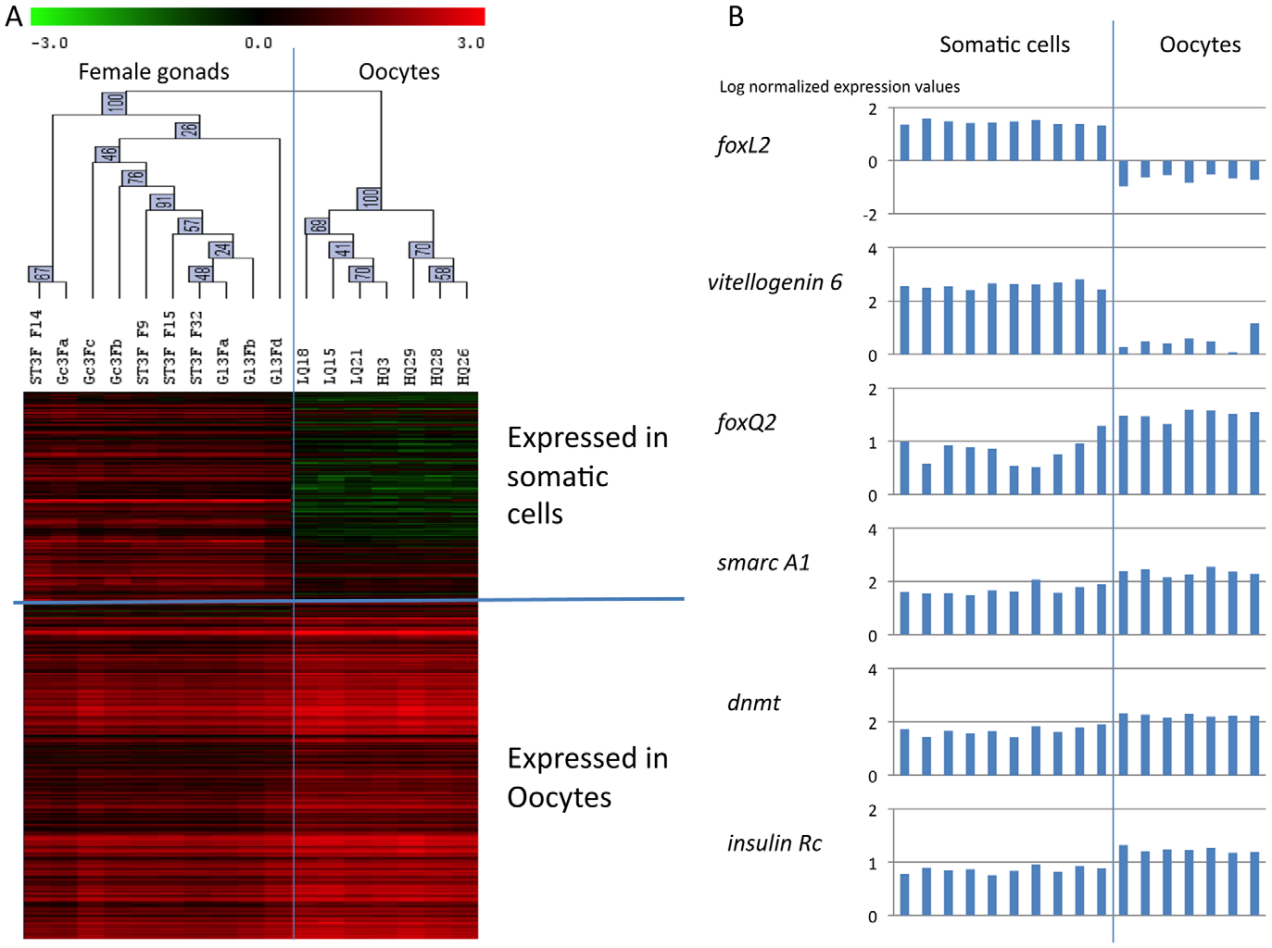
Germ cell versus somatic gene expression. A/Hierarchical clustering obtained using Pearson’s correlation on the 434 genes differentially expressed in stage 3 females gonads and stripped stage 3 oocytes (T test, p<0.01, adjusted Bonferroni’s correction; rows) and on the 17 transcriptomic profiles of stage 3 female gonads (columns). Two sample branches are observed, clustering the 4 individual stage 3 female gonads (St3F) and the 6 pools of stage 3 female gonads (Gl3F for individuals from site 1 and Gc3F for individuals from site 2) together on a first and the 7 stripped stage 3 oocytes (LQ and HQ) together on a second branch. Two clusters of genes are observed, grouping genes more expressed in whole stage 3 female gonads apart from genes more expressed in stripped stage 3 oocytes. Genes more expressed in whole stage 3 female gonads are predicted to be expressed by somatic tissues, in contrast with genes significantly more expressed in stripped oocytes. Color represents the transformed normalized Cy3 log value obtained for each sample. The variations in transcript abundance are depicted with a color scale, in which shades of red represent higher gene expression and shades of green represent lower gene expression. B/Expression profiles of 2 genes expressed in somatic cells: *forkhead box L2* (*foxL2*) [Genbank AM860211] and *vitellogenin 6* [Genbank AB084783]; and of 4 genes expressed in oocytes: *forkhead box Q2* (*foxQ2*) [Genbank AM865563], *SWI/SNF-related matrix-associated actin-dependent regulator of chromatin* A1 (*smarc A1*) [Genbank AM869433], *DNA methyltransferase* (*dnmt*) [Genbank CU994437], and *insulin receptor* (*insulin Rc*) [Genbank AJ535669] as measured by microarray analysis. A and B/Microarray data are expressed in log center reduced normalized values.

Oyster gonad is a mixed tissue including storage tissue, smooth muscle fibers and circulating hemocytes. In order to characterize the expression of genes involved in gametogenesis in oocytes or female somatic tissue, we studied the transcriptome of oocytes collected by stripping 7 mature females (stage 3) and compared them to the transcriptome of the 10 stage 3 female gonad samples (4 individuals and 6 pools from two different collection sites) (T test, P<0.01, adjusted Bonferroni’s correction). Among the 2,482 genes differentially expressed in both male and female gametogenesis, 434 were significantly differentially expressed between female gonad tissue and stripped oocytes. Genes for which more transcripts were found in whole stage 3 gonads are predicted to be expressed by female somatic tissues. When more transcripts were found in stripped mature oocytes, the genes are predicted to be expressed by female germ cells (Figure 4). Predicted localization of gene expression is provided in file S3.

Most of the genes expressed by oocytes showed an increase in expression along the gametogenetic cycle (clusters 2, 3, 7, 8 and 9, Figure 3, Table 1). In contrast, early expressed genes were mostly expressed by somatic cells in the gonadic area (clusters 1, 10, and 4, Figure 3, Table 1).

**Table 1:** Genes differentially expressed during gametogenesis and their expression in somatic cells or oocytes.

Cluster	Major expression stages	Number of genes	Female somatic cells	Oocytes
**1**	Stage 0	511	**110 (21.5%)**	2 (0.4%)
**2**	Stage 3 females	222	1 (0.4%)	**45 (20.3%)**
**3**	Stages 2–3 females	312	2 (0.6%)	**66 (21.1%)**
**4**	Stages 1–3 females	197	**34 (17.2%)**	6 (3.0%)
**5**	Stage 3 males	226	4 (1.8%)	**12 (5.3%)**
**6**	Stages 1–3 males	146	**8 (5.5%)**	4 (2.7%)
**7**	Stage 3 Males and Females	200	0 (0%)	**45 (22.5%)**
**8**	Stages 2–3 Males and Females	295	7 (2.4%)	**19 (6.4%)**
**9**	Stages 1–3 Males and Females	332	4 (1.2%)	**45 (13.6%)**
**10**	Stages 1–2 Males and Females	41	**19 (46.3%)**	1 (2.4%)

Female specific genes expressed by somatic tissues from stage 1 to stage 3 included genes known to be involved in sex differentiation such as *foxL2*, *vitellogenin-2* and –*6*, *membrane steroid binding protein 2* [Genbank CU989296], and *fatty acid synthase* [Genbank AM866384, AM866382]. Other genes expressed by somatic cells code for proteins characterizing microtubule formation and smooth muscle fibers such as Filamin-A, Filamin-C, Talin and Futsch found in cluster 10 and Titin, Myosin, Calponin, Transgelin [Genbank BQ426556], Collagen, Microtubule associated protein and Calmodulin found in cluster 1.

Genes expressed in oocytes were either sex specific (clusters 2, 3, 4) or found expressed in both male and female gonads (clusters 5, 6, 7, 8 and 9). Early expressed in the gonad during oogenesis, the *nanos homolog 3* was found specifically expressed by oocytes. In addition, genes involved in the maintenance of chromatin [Genbank FP002353, CU996984, CU999660, AM867260, CU683559, FP002000] and in transcriptional regulation and/or polarity information during tissue morphogenesis were expressed in oocytes such as genes coding for Arginine-glutamic acid dipeptide repeats proteins [Genbank FP000141, EX956437], Activating transcription factor-7 interacting protein [Genbank CU997418] Forkhead box Q2 [AM865563], Frizzled-1 [Genbank AM861216], and Protein strawberry notch homolog 1 [Genbank AM858733]. Finally, mature oocytes highly expressed genes coding for cell signaling molecules regulating cell proliferation and differentiation such as G proteins [Genbank AM855576, AM858539], a G protein-coupled receptor kinase [Genbank AM861820], a Putative molluscan insulin-related peptide receptor [Genbank AJ535669] and a Serine/threonine-protein kinase Pim-3 [genbank DV736298].

Some of the genes expressed in oocytes were also found in abundance in maturing male gonads suggesting that they are expressed by both male and female germ cells. Maturing germ cells express genes involved in chromatin condensation [Genbank CU999229, BQ427163], DNA replication control [Genbank AM862562, AM869187], DNA repair [Genbank AM865265, FP000995, AM862640, AM857512, AM868574], mitosis and meiosis regulation [Genbank CU685661, AM867561, FP000043, AM858065, AM861527, CU988908, AM858044] and the regulation of transcription [Genbank AM863854, AM869433]. Genes suggesting epigenetic regulation were highly expressed in mature germ cells (stage 3) such as the *polycomb protein suz12* [Genbank 863465] and *DNA (cytosine-5)-methyltransferase* [Genbank CU994437, CU999215].

### Technical and Biological Validation of Microarray Data

For real time qPCR technical and biological validation we selected 14 genes from the four main groups described above. They exhibit distinct and variable expression levels depending on the sex and the maturity of the oyster: higher expression in early gonad developmental stages (1 gene from cluster 1: *unc-93*, Genbank CU686276), or increasing expression over the course of gametogenesis in males (3 genes from cluster 5: *bindin*, Genbank EF219429; Genbank AM857898; Genbank AM864807; and 1 gene from cluster 6: *histone 4*, Genbank DW713865), in females (1 gene from cluster 3: *rcc1*, Genbank CU996984, 4 genes from cluster 4: *fox-L2*, Genbank AM860211; *vit-2*; Genbank CX069168; *vit-6*, Genbank AB084783; *nanos3*, Genbank CU994694), or in both males and females (3 genes from cluster 8: *cenpf*, Genbank AM862170; *mus309* Genbank AM859057; *prc1*, Genbank AM861527; 1 gene from cluster 9: *cyclin-B*, Genbank CU683817). The primer sequences used for real time qPCR are provided in file S4.

**Figure 5:**
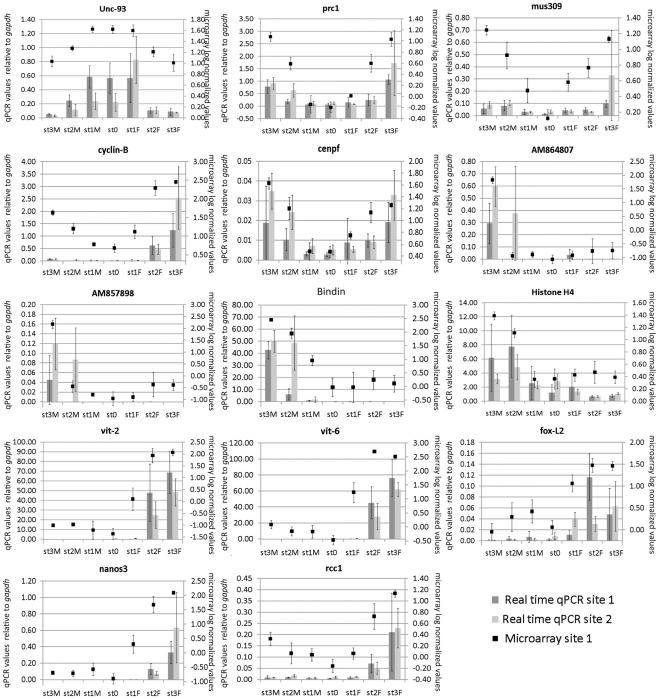
Expression profile of selected genes. Expression of 14 genes was measured in individual gonad samples from Site 1 (Locmariaquer, Brittany, France) and Site 2 (Baie des Veys, Normandy, France). Are displayed, expression profiles obtained by real time qPCR (histograms, left axe) and microarray (crosses, right axe). Vertical bars represent standard deviation for microarray data (doted line) and real time qPCR (plain line). mRNA expression levels of the 14 genes estimated by real-time qPCR are relative to *gapdh* (expressed in AU). Microarray data are expressed in log center reduced normalized values. Are displayed, expression profiles obtained for a gene more expressed in stage 0 and stage 1 : *unc-93 like protein* [Genbank 686276], three genes that increase in expression over the course of both male and female gametogenesis: *protein regulator of cytokinesis 1* (*prc1*) [Genbank AM861527], *bloom syndrome protein homolog* (*mus309*) [Genbank AM859057], *G2/Mitotic-specific cyclin-B* (*cyclin B*) [Genbank CU683817], *centromere protein F* (*cenpf*) [GenbankAM862170], four genes that increase in expression over the course of spermatogenesis: two unknown proteins [Genbanks AM864807 and AM857898], hypothetical protein *BRAFLDRAFT_118409* (*bindin*) [Genbank EF219429] and *histone H4* [Genbank DW713865], and five genes that increase in expression over the course of oogenesis : *vitellogenin-2* (*vit-2*) [Genbank CX069168], *vitellogenin-6* (*vit-6*) [Genbank AB084783], *forkhead box L2* (*foxL2*) [Genbank AM860211], *nanos homolog 3* (*nanos3*) [Genbank CU994694] and *regulator of chromosome condensation* (*rcc1*) [Genbank CU996984]. The patterns of transcripts abundance detected for these genes in the array and by real time qPCR showed extremely similar profiles (R^2^ = 0.80). For microarray, only Site 1 samples are shown. Developmental stage (St3: stage 3; St2: stage 2; St1: stage 1; St0: stage 0) and sex (M: male and F: female) are indicated at the bottom of each figure. Bars represent standard deviation.

For technical validation, gene expression was measured by real time qPCR in the same individual samples studied by microarray analysis (32 gonad samples from oysters from Site 1). The patterns of transcript abundance detected for these genes in the array and in real time qPCR showed extremely similar profiles. A mean coefficient of determination (R^2^) of 0.80 was obtained, data among the 14 genes ranging from R^2^ = 0.65 to R^2^ = 0.95 for the genes *mus309* and the *bindin precursor 5 repeat variant* respectively (Figure 5).

To assess the involvement of these genes in gametogenesis, the transcript abundance obtained by real time qPCR of individual oysters sampled in Brittany (site 1) was compared with the transcript abundance obtained for pools of RNA from oysters originating from another site (Site 2 in Baie des Veys, Normandy, France) (see Figure 5). Comparison of the levels of mRNA expression of the 14 genes in Site 1 and Site 2 revealed significant differences for 3 genes (ANOVA; p<0.05). The *centromere protein F* appeared more expressed in male and females individuals from Site 2; *bindin* appeared significantly more expressed in stage 2 males from Site 2 than in stage 2 males from Site 1; and *vitellogenin 2* expression was higher in females from Site 1 than in females from Site 2. Despite these differences in expression levels, all selected sex-specific genes produced exclusive signal intensities in the expected oyster pools and the higher gene expression at the expected stages.

To further assess the biological validity of our data, we performed a microarray analysis of three pools of stage 3 females from Site 2 and compared their transcriptomic profiles with the ones obtained from pools of individuals from Site 1. In the first instance, we repeated principal component analysis and found that all stage 3 female gonads clustered together regardless of their geographical origin. Furthermore, in the following clustering analyses, all stage 3 females (individuals and pools) clustered together (also observed in Figure 4). Finally, we performed a T test (p<0.01, adjusted Bonferroni’s correction) between the 3 pools of stage 3 females from each Site and did not find any differentially expressed gene. All together, these results confirm the biological validity of our findings.

## Discussion

Recent expression profiling studies using microarrays have provided great insight into the molecular mechanisms governing various complex physiological traits [37], [38], [39]. Among those, the unprecedented amount of information collected on gametogenesis, mitosis or meiosis of different eukaryotes such as yeast or nematode had a great impact on our understanding of sexual reproduction [40]. Microarrays have also been employed successfully to better understand the cellular and molecular events of the development of reproductive tissues and of embryogenesis of cattle [41], mouse [42], [43], [44], rat [45] and fish [46], [47], [48]. Here, we proposed to unravel some molecular mechanisms involved in sex differentiation and gametogenesis of a peculiar alternative hermaphrodite invertebrate, the Pacific oyster *Crassostrea gigas*.

We used an oligonucleotide microarray (Agilent) composed of 31,918 ESTs to characterize the transcriptome of oyster gonads at different developmental stages. The microarray employed in this study had previously been used to describe the transcriptome of various tissues of *C. gigas* [2] and the results were validated by showing a significant correlation of gene expression obtained by real time qPCR and microarrays. In the present study, we further validated our microarray data by measuring the expression of fourteen genes in 32 individual samples by real time qPCR. The high coefficient of determination obtained (80%) confirmed the high reliability of the microarray approach. This value was even shifted up when genes displaying low expression, and therefore less reliable Ct values in the correlation were excluded.

Gonad samples were collected from Pacific oysters originating from 3 different sampling sites, at the four stages of the yearly reproductive cycle of oysters [22], [23], [24], [25], [26], [27]. This sampling method was undertaken in order to compensate for a possible bias in transcriptome analysis due to singularity of a single population within a precise environmental context. The results were mainly obtained from studying transcriptomic profiles from site 1 individuals [49], and show that the transcriptome of samples collected from different geographical locations are not significantly different regarding gametogenesis. Thus, we compared the transcriptomes of gonads from site 1 and site 2 using both microarray and real time qPCR analyses. The high correlation between geographical locations confirmed that the expression profiles observed are real features of gametogenesis in oysters without significant influence of the sampling site.

Our analysis provided lists of genes expressed in male and female gonads, genes that increase in expression along the gametogenetic cycle, genes expressed in the flagella structure of spermatozoids, genes expressed in oocytes and genes expressed by female somatic cells. Most importantly, cross-referencing these lists of genes allowed us to identify potential markers of early sex differentiation in *C. gigas* oyster, a singular alternative hermaphrodite mollusk. We also provided new highly valuable information on genes specifically expressed by mature spermatozoids and mature oocytes.

### Sex Differentiation

In order to identify new candidate genes involved in sex differentiation, we screened for male- and female-specific genes and genes expressed early at the beginning of gametogenesis. We found a higher number of early expressed female-specific genes than male-specific genes. The reason for this may be directly related to the greater transcription occurring in female germ cells compared to male germ cells [50]. Indeed, oocytes provide most of the metabolic resources and information required for the early development of zygotes. They accumulate as mRNAs and proteins within the oocyte cytoplasm during vitellogenesis [50].

Among the male-specific genes, we identified the previously characterized *bindin* precursor 1, 2, 3, 4 and 5 variants [35]. Bindin is an insoluble protein necessary for sperm-egg bonding during fertilization. The protein is stored in the acrosomal granule of spermatozoids and is released by exocytosis when sperm contacts the egg surface [30], [34], [35], [51]. One hundred and forty six genes showed a gradual increase in expression from stage 1 to stage 3. Such an increase is likely to reflect the gradual proliferation and differentiation of germ cells within the sample. These genes including *meiotic recombination protein DMC1/Lim15 homolog, synaptonemal complex protein 1* were mainly involved in the meiotic phase of spermatogenesis or coding sperm proteins such as the Histone H4 with egg coat binding capabilities [52]. Among the genes expressed from stage 1, we particularly noticed the histone methyltransferase *dpy-30*. *dpy-30* is essential for hermaphrodism dosage compensation, epigenetic regulation and male development in *C. elegans* [53]. Thus, *dpy-30* constitutes an attractive new candidate gene for the regulation of sex differentiation in oysters.

Among the female specific genes, we identified *forkhead box L2* (*foxL2*), a highly conserved female specific transcription factor [31], and *vitellogenin*, a female specific glycoprotein previously identified as being necessary for building up the oocyte in oysters [54]. Our data showed that both *foxL2* and *vitellogenin* were expressed by somatic cells. In mammals, *foxL*2, expressed in follicular cells, is the earliest known marker of ovarian differentiation [55], [56]. Genes coding for Lipoprotein lipase and Pancreatic lipase-related proteins were also found highly expressed, a result similar to what had been observed in the European sea bass (*Dicentrarchus labrax*) and the Yesso scallop (*Patinopecten yessoensis*) respectively [57], [58]. Genes involved in blocking meiosis at prophase I when kinetochores assemble on the centromeres were also highly expressed in females and absent in males. For example, the Mitotic apparatus protein p62 binds to condensed chromosomes at prophase of meiosis I; Condensin-2, expressed in oocytes, is involved in maintaining the rigidity of chromosomes in prophase; G2/mitotic specific Cyclin B3 is expressed until early meiotic prophase and is required for female fertility in *Drosophila* [59]; The protein NudE-like interacts with kinetochores at the centrosome [60]. Two Tetraspanins (CD151 and CD63) were also significantly more expressed in females than males. This family of transmembrane proteins often acting as scaffolding proteins participates in a variety of cellular processes, such as cell activation, adhesion or differentiation though their exact function remains unclear. Interestingly, the Histone H1 isoform/protein B4 was also identified from stage 1. This Histone H1 isoform is known to control gene expression during oogenesis through perturbation of chromatin structure and is involved in the maintenance of proximal germ cells by supporting their proliferation [61]. In addition, *nanos homolog 3 was* specifically expressed in oocytes. In hermaphrodite *C. elegans*, Nanos-3 controls the sperm-oocyte switch as revealed in Nos-deficient individuals, producing excess sperm and no oocytes [62]. *nanos homolog 3* may also be involved in female germ cell differentiation.

Overall, when a function could be assigned to an EST, the male or female-specificity of the gene expression was confirmed by comparison with the corresponding gene functions in other species. Often, the gene function was related to sex differentiation such as *dpy-30* in males and *fox-L2* and *nanos-homolog 3* in females. The sex- and gonad-specific expression of the remaining non-annotated genes constitutes a valuable tool towards the assignment of gene function. The future characterization of these newly identified male and female specific genes should provide great insight into oyster sex differentiation.

We initially hoped to use PCA to discriminate between male and female stage 0 oysters and to identify genes involved in early sex differentiation. However, no difference was observed between the eight stage 0 gonads analyzed. Therefore, the future sexual development of these gonads could not be predicted from this PCA analysis. Principal component analysis revealed that differences between males and females increased overtime, from stage 1 gonads to stage 3 gonads, suggesting that sex differentiation takes place sometime before. Interestingly, some of the studied stage 0 gonads were found to express male specific (*bindin*) or female specific genes (*foxL2*, a *pancreatic lipase related proteins* and *cd63*), suggesting that sex differentiation already took place within these individuals although it was not possible to sex them using histology. We did not observe mitosis and cell proliferation within these individuals by histological methods. However, we performed cytology by observing a single transverse section of the gonad collected in the middle of the organ. Heterogeneity in the development of germ cells at different levels of the gonad has been observed and this may explain that some sex specific genes are found expressed when gene expression is measured on the whole gonad. Thus, the individuals presumed to be at stage 0 according to histological characterization, may biologically correspond to early stage 1 individuals. Few differences were observed between the transcriptomic profiles of undifferentiated stage 0 and sexed stage 1 individuals. Differences seem to fit with germ cell proliferation and the onset of meiosis in stage 1. The mitosis/meiosis and sperm/oocyte decision may take place during the same time frame in the oyster gonad. In *C. elegans*, *fbf* (PUF family) and *gld-3* genes control both the decision to leave the mitotic cell cycle and enter meiosis and to achieve the switch from spermatogenesis to oogenesis [63], [64], [65], [66]. In vertebrates, the first sexual differences in male and female gonads is their timing of onset into meiosis, regulated by retinoic acid [67]. Similarly, in oysters, overlapping pathways may control mitosis/meiosis and sperm/oocyte switches.

In oysters, the gonad consists of numerous tubules, invaginated in storage tissue and mantle. During gonad development, tubules develop at the expense of the storage tissue [25], [28], [29]. When dissecting gonad at the earlier stages (0 and 1), we collected a tissue commonly designed “mantle-gonad”, surrounding the visceral mass and consisting in a mix of conjunctive, germinal and other somatic cells. The low number of germ cells present in stages 0 and 1 samples renders the identification of genes specifically expressed early in gonad development difficult [22]. Therefore, a large part of the genes more expressed in stage 0 and stage 1 gonads (cluster 1) was likely to represent somatic cells. Indeed, numerous muscle fibers specifying genes, such as those encoding the calcium binding proteins Calponin and Transgelin, the actin-depolymerising factor and the molecular spring Titin, were found highly expressed in mantle and adductor muscle [2]. One hundred and twelve genes from cluster 1 were significantly less expressed in stripped oocytes, further suggesting that they were expressed by somatic cells. Only two genes (Genbank CU683682 and FP006096) from this cluster were significantly more expressed in oocytes than in somatic cells (table 1). The germ cell specific expression makes them extremely interesting despite the lack of homologies to any other known genes. Identifying genes specifically expressed by germ cells at the beginning of gametogenesis and sex differentiation will require to enrich samples in germ cells, which might be solved by gene expression analysis of laser capture microdissected cells [26].

### Spermatogenesis

We identified 372 genes potentially involved in spermatogenesis. Two hundred and twenty six genes were expressed strictly in stage 3 males and may include genes involved in spermatozoid and mature sperm formation. A high expression of genes involved in ubiquitination and proteasomal degradation of targeted E3 ubiquitin proteins is observed in stage 3 individuals (*ariadne-1, march3, kelch-like proteins, adaptor of E3 ubiquitin ligase*). Ubiquitin targeting and proteasome take an important place in late spermatogenesis stages. Indeed, an ubiquitin-dependent sperm quality control has been observed in mammals [68]. A similar process may take place in oysters before sperm is released into the ocean.

Investigation of tissue expression of the genes involved in spermatogenesis revealed that a high proportion of these genes were also highly expressed in gills and labial palps, suggesting functional similarities between these tissues. The functions of these genes were related to flagella and cilia structure and movement reflecting the common transcriptomic features of spermatozoa flagella with gills and labial palps ciliated epithelia. For example, genes encoding Dynein and Kinesin-like proteins were found. The sperm surface protein sp17 that used to be described as a sperm specific protein, plays also a regulatory role in human somatic ciliated cells [69].

### Oogenesis

In females, several of the genes with increasing transcription patterns from stage 2 or 3 code for regulators of gene expression, such as genes encoding for Aryl hydrocarbon receptor nuclear translocator protein, CC4-NOT transcription complex subunit 7, regulators of chromosome condensation, and Protein strawberry notch homolog 1. The cell cycle blockade in oocytes is suggested by the high expression of genes coding for the Nucleoporin 53, that sequester MAD2 to control cell cycle, and Cep57, a centrosomal protein required for microtubule attachment to centrosomes. Among the genes highly expressed in mature female gonads, we noticed the presence of a gene coding for the Methyl-CpG binding domain protein 2, involved in binding methylated DNA and repressing transcription of methylated gene. Its high expression suggests an epigenetic transfer of information by oocytes across generations. A significant part of these genes might represent maternal mRNA, known to be stocked in oocytes during oogenesis and maternally transmitted to embryos before the start of embryonic transcription. The high gene expression coding the protein LSM14 homolog A may illustrate the translational repression of mRNA and the formation of P bodies regulating maternal mRNA expression [70], [71]. We also showed an increase in expression of *maternal embryonic leucine zipper kinase* (*MELK*), with unknown function in oysters but involved in embryonic development in vertebrates [72]. Finally, expressed by stage 3 female oocytes, we identified Forkhead box Q2 involved in early patterning in embryos [73] and Frizzled-1 that interacts specifically with Wnt and regulates axial patterning [74], [75].

### Immunity

The identification of immune relevant genes such as genes coding for Complement C3, a PZP-like alpha-2-macroglobulin domain containing protein, Small proline rich protein 3, Chitotriosidase, Superoxide dismutase and CD109 in mantle and gonad suggests that they might be expressed by tissue-bathing hemocytes infiltrated in the mantle and protecting the gonad. In marine bivalves, immune genes have previously been reported highly expressed in gills [76], [77] or in gonads [78]. In some instance, further investigations revealed their mRNA expression in tissue-infiltrating hemocytes [79] at possibly higher levels than in circulating hemocytes [80]. The function and site of expression of these immune related genes highly expressed in gonads and mantle should be further explored, especially in the context of oyster summer mortalities [13] one of the current major concern of oyster aquaculture in the world.

### Conclusions

The most significant outcome of our study is the identification of transcripts that improve our understanding of gametogenesis in the Pacific oyster and produce lists of relevant candidate genes for further studies. Here we report temporal variation of gene expression during oyster gonad differentiation and development. In addition to genes preferentially expressed at each differentiation stage and for each sex, we compared the data with a dataset of gene expression in somatic tissues and in oocytes and identified subsets of genes specifically expressed in oocytes, somatic cells and in flagella and cilia structures. Furthermore, to reveal new clues in determining the pathways involved in sex differentiation in *C. gigas*, a facultative protandrous alternative hermaphrodite, we identified genes specifically expressed in either males or females. Their expression in undifferentiated individual gonads allowed a better characterization of the time frame when sex differentiation may take place. Only 1,085 of the 2,482 genes significantly altered in males and/or females over the course of gonadogenesis were annotated. Thus, our discussion was limited to these proteins for which the function in oysters can be speculated. Our dataset represents a valuable and solid base for further research investigating the molecular mechanisms involved in oyster gonad differentiation and development. The actual *in vivo* function of the new candidate genes potentially involved in sex differentiation will obviously require the development of gene knock-down strategies such as RNA interference, a functional assay that was recently found to operate in oyster [33].

## Materials and Methods

### Animal Sampling

The collection sites were designed as zone specific for marine culture in the French public register for marine coastal land (“Cadastre conchylicole”) so that no specific permits were required for our field study. In south Brittany, the field study was done along the coast in specific zones owned by the Ifremer Institute. The oyster *Crassostrea gigas* is not an endangered or protected species.

Oysters were sampled 8 times between November 2008 and September 2009 in Site 1 (Locmariaquer, Brittany, France), 9 times between January 2008 and October 2008 in Site 2 (Baie des Veys, Normandy, France) and once in July 2010 in Site 3 (Argenton, Brittany, France) in marine coastal areas specifically dedicated for marine culture and owned by the Ifremer Institute. For each site, at the beginning of the reproductive season oysters were 1.5 years old. At each sampling date, oyster gonads were immediately dissected. Gonad tissues from sites 1 and 2 were sampled for each oyster for RNA extraction and also fixed for histological analysis and sex determination. Sample of gonads from site 3 were fixed for histological analysis and immediately stripped for oocytes collection. Gonad developmental stage and sex were determined by histological methods according to the 4 stages previously described [24], [25]. For total RNA isolation, individual samples were homogenized in Tri-reagent™ (Sigma) (50 mg/1 mL) and stored at –80°C for total RNA extraction.

### RNA Extraction

Samples in Tri- reagent™ (Sigma) were solubilized using a syringe (0.9 mm). Total RNA was then isolated using Extract-all (Eurobio) procedure. RNA quality and integrity was controlled as previously described [2] on the Agilent bioanalyzer using RNA nanochips and Agilent RNA 6000 nanoreagents (Agilent Technologies, Waldbronn, Germany). RNA concentrations were measured at 260 nm using an ND-1000 spectrophotometer (Nanodrop Technologies) using the conversion factor 1 OD = 40 µg/mL RNA. Samples were stored at –80°C until further use.

### cDNA Microarray

#### RNA amplification, labeling and hybridization

Four individual gonad samples from Site 1 were prepared for microarray analysis for each gonad developmental stage and sex, except for stage 0 for which RNA from 8 individual gonads were sampled. Three pools of RNA from 6 stage 3 females from Site 2 and 3 pools of RNA from 6 stage 3 females from Site 1 were also processed for biological validation of microarray data (see Results). An additional 7 individual samples of oocytes from Site 3 were prepared for microarray analysis. Two hundred nanograms of total RNA was indirectly labeled using the Low Input Quick Amp labeling kit (Agilent) according to the manufacturer’s instructions. Qiagen’s RNeasy mini spin columns were used for purifying amplified RNA (aRNA) samples. After purification, the labeled aRNA concentration was between 200 and 500 ng/µL and dye incorporations between 20 and 50 pmol/ µg aRNA. RNA amplification and dye incorporation rates were controlled using an ND-1000 spectrophotometer (Nanodrop Technologies). Hybridization was performed using the Agilent’s Gene expression hybridization kit (5188–5242) with 1.65 µ g of aRNA samples labeled with Cy3. Subsequently, slides were washed with Gene expression wash buffer solution (5188–5327; Agilent Technologies) and Stabilization and Drying solutions (5186–5979; Agilent Technologies). Slides were then scanned on an Agilent Technologies G2565AA Microarray Scanner system at 5 µm resolution.

#### Correction and normalization

Raw data extraction and normalization were conducted with Agilent Feature Extraction software 6.1 using the default/recommended normalization methods. A matrix of gene expression levels was generated, where each row corresponds to a different gene and each column to one oyster gonad sample. The expression level of each gene was then logarithmically transformed and centered (relative to zero) as in [81], so that relative variations rather than absolute values were used for interpretation.

#### Data analysis

We initially applied a principal component analysis (PCA), using GeneANOVA software [82], to assess the internal consistency of different transcriptional data sets from the same gonad developmental stage. The proportion of variance for each principal component and the cumulative variance were obtained. The three components with the highest proportion of variance were used to draw a 3D scatter plot (XLStat; Addinsoft) organizing the 32 oyster transcriptomes from site 1 along the principal components.

To compare male and female transcriptomes, we used student’s T tests with a p-value exceeding 99% confidence (i.e. p<0.01) and an adjusted Bonferroni’s correction on all male vs all female oyster gonads (stage 1, 2 and 3 together) using TMeV 4.6.0 software [83], [84].

Then, we identified statistically significant differentially expressed transcripts within male and female time-course. A one-way ANOVA parametric test was used to investigate the significance of the factors stage and sex using a p-value cut-off of 0.01 and an adjusted Bonferroni’s correction using TMeV 4.6.0 software [83], [84] as previously described [2]. Cluster analysis was employed to further demarcate the expression patterns occurring during gonad development. Hierarchical clustering and K means clustering were performed using TMeV on the statistically significant transcripts described previously to cluster transcripts based on similarity of expression between oyster gonads [83], [84]. Hierarchical clustering was used to group experimental samples together based on similarity of the overall experimental expression profiling.

Gene expression localization was inferred from the results of a student’s T test with a p-value exceeding 99% confidence (p<0.01) and an adjusted Bonferroni correction on all 7 stripped stage 3 oocytes samples vs all 4 individuals and 6 pools of stage 3 female gonads using TMeV 4.6.0 software [83], [84].

### Real Time Quantitative PCR

A first set of samples, constituted of RNA samples of individuals from Site 1 prepared for microarray analysis, were processed for real time qPCR. A second set of samples was prepared as follow with individuals from Site 2: RNA samples from 6 individuals were pooled for each gonad developmental stage and sex. Four pools were prepared for each condition leading to a total of 28 analyzed pools of oyster gonads. All RNA samples of individuals from Site 1 and pools from Site 2 were treated with DNAse I (1 U/µg total RNA, Sigma) according to the manufacturer’s instructions and finally washed twice in 500 µL of Ethanol 95% prior to dilution in 10 µL of RNAse/DNAse-free water. RNA quality and quantity were assayed as described above. Two hundred and seventy ng of total RNA were reverse-transcribed and amplified by real time qPCR. Amplification reactions contained 1× QuantiTect SYBR Green PCR Buffer (Qiagen), 5 ng of DNA template, and 900 nM of each primer in a final volume of 15 µL. Each run included a positive cDNA control (one sample of the present experiment analyzed in each amplification plate), negative controls (each total RNA sample with DNAse I treatment) and blank controls (water) for each primer pair. The comparative threshold cycle (C_T_) method was used to quantify target gene copy number in the gonad DNA sample relative to that of an endogenous control gene. We used geNorm on Ct to determine the expression stability (M value) [85] of potential control genes [2] and validated the selection of *gapdh* for real time qPCR. PCR efficiency (E) was estimated for each primer pair by determining the slopes of standard curves obtained from serial dilution analysis of a reference cDNA sample to ensure that E ranged from 95 to 100%. Primer names, accession numbers, PCR efficiencies and sequences are listed in file S4.

## Supporting Information

File S1 **List of sex-specific expressed genes.** This table provides the list of genes differentially expressed between all male vs all female gonad samples (T test; p<0.01 with adjusted Bonferroni’s correction). ID_Ref: Identity of the spot on the microarray; Genbank: Genbank accession number; Description: description as uploaded from Sigenae (http://www.sigenae.org); Mean Males: mean of log normalized expression values of stage 1, 2 and 3 individual males; StDev Males: standard deviation of log normalized expression values of stage 1, 2 and 3 individual males; Mean Females: mean of log normalized expression values of stage 1, 2 and 3 individual females; StDev Females: Standard deviation of log normalized expression values of stage 1, 2 and 3 individual females; Abs t value: Absolute t value; df: degrees of freedom; Raw p value; Adj p value: Adjusted p value.
(XLS)

File S2 **List of genes differentially expressed over the course of male and/or female gametogenesis.** This table provides the list of genes differentially expressed over the course of male and/or female gametogenesis (ANOVA; p<0.01 with adjusted Bonferroni’s correction). Genes have been grouped in 10 clusters (K means clustering using pearson’s correlation). ID_Ref: Identity of the spot on the microarray; Genbank: Genbank accession number; Description: description as uploaded from Sigenae (http://www.sigenae.org); Cluster: Cluster obtained from K means clustering; Tissue enriched expression: Tissue expression as observed in Dheilly et al. [2]; High expression: High expression values in somatic tissues; Mean St0: mean of log normalized expression values of stage 0 individuals: StDev St0: Standard deviation of log normalized expression values of stage 0 individuals; Mean St1 M: mean of log normalized expression values of stage 1 male individuals; StDev St1 M: Standard deviation of log normalized expression values of stage 1 male individuals; Mean St2 M: mean of log normalized expression values of stage 2 male individuals; StDev St2 M: Standard deviation of log normalized expression values of stage 2 male individuals; Mean St3 M: mean of log normalized expression values of stage 2 male individuals; StDev St3 M: Standard deviation of log normalized expression values of stage 3 male individuals; Mean St1 F: mean of log normalized expression values of stage 1 female individuals; StDev St1 F: Standard deviation of log normalized expression values of stage 1 female individuals; Mean St2 F: mean of log normalized expression values of stage 2 female individuals; StDev St2 F: Standard deviation of log normalized expression values of stage 2 female individuals; Mean St3 F: mean of log normalized expression values of stage 2 female individuals; StDev St3 F: Standard deviation of log normalized expression values of stage 3 female individuals; F ratio; SS(groups): Sum of square between groups; SS (error): Sum of squares of error; df (groups): degrees of freedom for groups; df (error): degrees of freedom for error; raw p value; Adj p value: Adjusted p value.
(XLSX)

File S3 **List of genes differentially expressed between whole stage 3 female gonads and stripped stage 3 oocytes.** This table provides the list of genes differentially expressed between whole stage 3 female gonads and stripped stage 3 oocytes (T test; p<0.01 with adjusted Bonferroni correction). ID_Ref: Identity of the spot on the microarray; Genbank: Genbank accession number; Description: description as uploaded from Sigenae (http://www.sigenae.org); Cluster: Cluster obtained from K means clustering; Tissue enriched expression: Tissue expression as observed in Dheilly et al. [2]; Mean gonad: mean of log normalized expression values of individual stage 3 females and pools of stage 3 females; StDev gonad: standard deviation of log normalized expression values of individual stage 3 females and pools of stage 3 females; Mean oocytes: mean of log normalized expression values of stripped stage 3 oocytes; StDev oocytes: Standard deviation of log normalized expression values of stripped stage 3 oocytes; Abs t value: Absolute t value; df: degrees of freedom; Raw p-value; Adj p-value: Adjusted p-value.
(XLS)

File S4 **primer sequences.** The expression of 14 genes has been measured by real time qPCR. For each gene, are provided: the Genbank accession number (Genbank Acc), the description as uploaded from Sigenae (http://www.sigenae.org), the cluster where the gene was identified (cluster), its main expression profile (expression; st0: stage 0; M+F: males and females; M: males; F: females), the primer sequence (seq), its length (length), Reverse or Forward (L vs R), its melting temperature (Tm), the percentage of GC (GC%), the product size and the primer pair efficiency (Efficiency (%)).
(XLS)
